# A Specific Primed Immune Response in *Drosophila* Is Dependent on Phagocytes

**DOI:** 10.1371/journal.ppat.0030026

**Published:** 2007-03-09

**Authors:** Linh N Pham, Marc S Dionne, Mimi Shirasu-Hiza, David S Schneider

**Affiliations:** Department of Microbiology and Immunology, Stanford University, Stanford, California, United States of America; University of Minnesota, United States of America

## Abstract

*Drosophila melanogaster,* like other invertebrates, relies solely on its innate immune response to fight invading microbes; by definition, innate immunity lacks adaptive characteristics. However, we show here that priming *Drosophila* with a sublethal dose of Streptococcus pneumoniae protects against an otherwise-lethal second challenge of *S. pneumoniae.* This protective effect exhibits coarse specificity for S. pneumoniae and persists for the life of the fly. Although not all microbial challenges induced this specific primed response, we find that a similar specific protection can be elicited by *Beauveria bassiana,* a natural fly pathogen. To characterize this primed response, we focused on S. pneumoniae–induced protection. The mechanism underlying this protective effect requires phagocytes and the Toll pathway. However, activation of the Toll pathway is not sufficient for priming-induced protection. This work contradicts the paradigm that insect immune responses cannot adapt and will promote the search for similar responses overlooked in organisms with an adaptive immune response.

## Author Summary

Due to the common practice of vaccination and prominence of AIDS, people are already aware of the distinction between adaptive and innate immunity without realizing it. All organisms have an innate immune response, but only vertebrates possess T cells and the ability to produce antibodies. It has been a long-standing assumption that invertebrate immune systems are not adaptive and respond identically to multiple challenges. In this study, we demonstrate that the fly innate immune response adapts to repeated challenges; flies preinoculated with dead Streptococcus pneumoniae are protected against a second, otherwise-lethal dose. Although the underlying mechanisms are likely to be very different, this primed response is reminiscent to vaccine-induced protection in that it exhibits coarse specificity (dead S. pneumoniae only protects against itself), persists for the life of the fly and is dependent on phagocytic cells. This result prompts the obvious question of whether the innate immune system of vertebrates shares a similar biology. Such a finding is of particular interest since immunocompromised individuals only possess an innate immune system.

## Introduction

Immune responses are typically characterized as being either adaptive or innate. Adaptive immunity, which requires T and B cells, is specific, has memory, and is generally considered to be restricted to vertebrates. In contrast, the innate immune response is thought to act naïvely to each encounter with a pathogen [1,2]. Innate immunity depends on the recognition of broadly conserved molecular moieties and exhibits only weak specificity, such as the ability to distinguish between different structural classes of peptidoglycan [3]. However, recent work suggests that the invertebrate innate immune response may exhibit adaptive characteristics (reviewed in [4] and [5]).

Functional immune adaptation can be defined most broadly as any case where an immune response differs between a first and second challenge. The simplest form involves the immune system remaining activated after an initial challenge. This sort of response has long been known in invertebrates as shown by Hans Boman and coworkers [6]. They found that antibacterial activity in *Drosophila* hemolymph persists after bacterial challenge and can provide protection against subsequent challenges. More recently, Moret et al. [7] found a similar persistence of humoral antibacterial activity in mealworms.

More complex adaptive phenomena have also been observed in arthropods; for example, flour moths [8] and *Daphnia* [9] possess strain-specific immunity that is passed from a mother to her offspring. The molecular mechanisms underlying the maternal transfer of strain-specific protection have not been characterized. Specific memory has also been examined in cockroaches [10] and bumblebees [11]. In both insects, the initial immune activation is nonspecific and confers protection against many types of challenges. However, both cockroaches and bees are also able to mount long-term specific protection: a priming dose of a particular species of bacteria only protects against that species (or class of species in the case of bumblebees). From this work, it seems that innate immunity possesses the adaptive characteristics of specificity and memory; unfortunately, the animals used in past studies have not been amenable to deeper analysis. We examined the well-understood *Drosophila* innate immune response for specificity and memory because this model organism would give us genetic and physiological assays to dissect adaptive aspects of innate immunity.

*Drosophila* has been proved to be a powerful model organism to study innate immunity [1,2]. The *Drosophila* innate immune response has three effector mechanisms: the humoral response, melanization, and the cellular response [1,2]. The humoral immune response involves the secretion of soluble factors, such as antimicrobial peptides (AMPs), into the hemolymph following immune activation. Melanization is the process whereby melanin is deposited at wound sites and parasite surfaces, resulting in the release of toxic reactive oxygen species. The cellular immune response consists of hemocytes that phagocytose, encapsulate, and kill invading microbes, much like vertebrate macrophages. These mechanisms depend in various ways on pathogen detection via the Toll or imd signaling pathways [1,2,12,13].

We found that S. pneumoniae–primed flies are protected against a subsequent lethal challenge with S. pneumoniae. This response is specific for S. pneumoniae and persists for the life of the fly. In this paper, we demonstrate that the Toll pathway, but not the imd pathway, is required for this protective effect. Notably, activation of the Toll pathway is not sufficient to elicit a primed response. We show that AMPs are not involved and that phagocytes are the critical effectors of the primed response. Taken together, we demonstrate that the *Drosophila* primed response is specific and persists for the life of the fly. We identified a signaling pathway required for the process and ascertained which branch of the fly immune response is responsible for the primed response.

## Results/Discussion

### The *Drosophila* Immune Response Can Adapt

**Figure 1:**
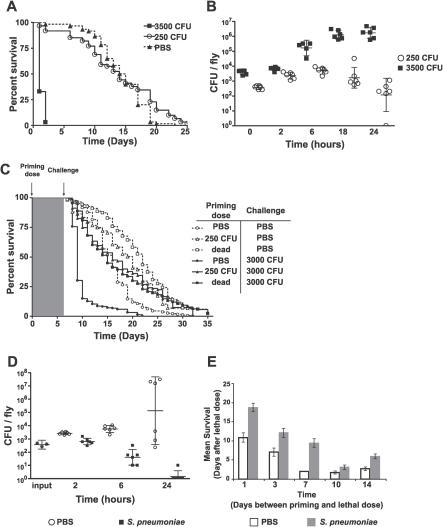
Protection from a Priming Dose of S. pneumoniae Persists for the Life of the Fly (A) Survival curves of flies injected with PBS (triangles, *n* = 60) or 250 (circles, *n* = 60) or 3,500 (squares, *n* = 65) CFU S. pneumoniae. *p* < 0.001, comparing 3,500 CFU to the other treatments (log-rank analysis). (B) Flies were injected with 250 (circles) or 3,500 (squares) CFU of S. pneumoniae. Bars represent geometric means of bacterial load with 95% confidence intervals. (C) Flies were primed on day 0 with PBS (circles), 250 CFU of S. pneumoniae (triangles), or dead S. pneumoniae (squares). One week later, flies were injected again with either PBS (open shapes) or 3,000 CFU S. pneumoniae (filled shapes). Naïve PBS-injected flies challenged with 3,000 CFU of S. pneumoniae die significantly faster than flies primed with 250 CFU of S. pneumoniae or dead S. pneumoniae (*p* < 0.0001, log-rank test). Dotted lines correspond to double injection controls. *n* = 158 to 228 for each condition. (D) Flies were injected with PBS (circles) or dead S. pneumoniae (squares) on day 0 and challenged 1 wk later with 400 CFU of S. pneumoniae. Bars represent geometric means of bacterial load with 95% confidence intervals. (E) Flies were primed on day 0 with PBS (open bars) or dead S. pneumoniae (filled bars) and challenged with 5,000 CFU of S. pneumoniae on the indicated days. Mean survival with standard error values are plotted. *n* = 37 to 49 for each condition. At each time point, the survival curves differ significantly. See Figure S1 for log-rank analysis and individual survival curves.

We found that previous exposure to S. pneumoniae permanently alters the fly's response to this bacterium. S. pneumoniae is a Gram-positive encapsulated bacterium that is the causative agent of otitis media, pneumonia, and meningitis [14]. The injection of 3,000 colony-forming units (CFU) directly into *Drosophila* hemolymph is normally lethal, killing the fly within 2 d (Figure 1A), and death is correlated with bacterial proliferation (Figure 1B). However, flies primed with a sublethal dose of bacteria were protected against a lethal challenge of S. pneumoniae administered 1 wk later (Figure 1C). Flies primed with S. pneumoniae and challenged with a lethal dose died at the same rate or slower than wounded controls. A priming dose of dead heat-killed S. pneumoniae was also sufficient to protect flies against a subsequent lethal challenge. Priming flies with S. pneumoniae thus induces long-term changes in the fly immune response, conferring protection against an otherwise-lethal challenge.

We considered two scenarios that could explain enhanced survival in S. pneumoniae–primed flies. First, bacterial numbers may not differ between naïve versus primed flies, and primed flies might survive the stress of the infection better than naïve flies. Second, S. pneumoniae–primed flies could kill the bacteria faster, and bacterial clearance would correlate with enhanced survival. To distinguish between these two possibilities, we examined bacterial load in naïve versus primed flies. Flies were injected with a priming dose of either dead S. pneumoniae or phosphate-buffered saline (PBS) 1 wk prior to a challenge of 400 CFU S. pneumoniae. This dose was chosen to emphasize the difference between naïve and primed flies; 400 CFU is the lowest dose that is lethal to naïve flies but not S. pneumoniae–primed flies (unpublished data). Within 1 d, S. pneumoniae–primed flies had killed almost all of the *S. pneumoniae,* whereas naïve flies still contained bacteria (Figure 1D). This result indicates that the survival difference between naïve and S. pneumoniae–primed flies results from different rates of S. pneumoniae killing.

Functionally, immunological memory is characterized by a more effective immune response upon repeat exposure that persists for the life of the animal. Although the best-described model for immune memory involves T and B cells and recombination-derived variation of receptors, we note that the definition of memory is independent of mechanism and immune memory could arise in a variety of ways. We demonstrated that preinoculation with dead S. pneumoniae alters the fly immune response such that it is more effective against subsequent challenges with *S. pneumoniae.* To determine how long these immune changes persist in the fly, we next varied the length of time between the priming and challenge dose. Flies primed with dead S. pneumoniae on day 0 were challenged between 1 and 14 d later with a lethal dose of *S. pneumoniae. S. pneumoniae*–primed flies always died significantly more slowly than naïve PBS-injected flies (Figures 1E and S1). An interval of 2 wk between the priming dose and challenge dose was the longest time we could assay; at 3 wk postpriming, the flies are actually 5 wk old and die from the stress of wounding alone (unpublished data). In summary, protection due to a priming dose of S. pneumoniae was detectable within 24 h and persisted for the life of the fly, or as long as we could assay survival differences.

### The *Drosophila* Immune Response Possesses a Surprising Degree of Specificity

**Figure 2:**
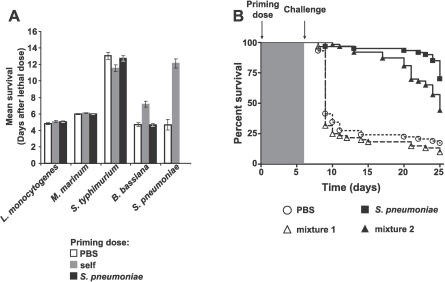
The S. pneumoniae–Induced Primed Response Is Specific for S. pneumoniae (A) Flies were injected with a priming dose of PBS (white bars), the same bacteria used for the lethal challenge (gray bars), or S. pneumoniae (black bars). Bacteria used for lethal challenges are indicated below the graph. Mean survival with standard error values are plotted. *n* = 57 to 128 for each condition. *p* < 0.001 for the indicated set of bars (log-rank test). See Figure S2 for individual survival curves and log-rank analysis. (B) Flies were primed on day 0 with PBS (circles, *n* = 59), dead S. pneumoniae (squares, *n* = 60), mixture 1 (dead *E. coli, M. luteus,* and *B. bassiana;* triangles, *n* = 59), or mixture 2 (dead *E. coli, M. luteus, B. bassiana,* and *S. pneumoniae;* triangles, *n* = 60) and challenged 1 wk later with 3,000 CFU of S. pneumoniae. Log-rank analysis indicates that curves corresponding to PBS and mixture 1 (dotted lines) are significantly different from those for flies primed with S. pneumoniae or mixture 2 (solid lines) (*p* < 0.001).

To explore the specificity of this immune response, we asked whether other pathogens can induce a protective response against themselves. We chose a broad range of microbes pathogenic to wild-type *Drosophila,* including a Gram-negative bacterium, a Gram-positive bacterium, a Mycobacterium, and a natural fungal pathogen [15–18]. Priming doses of heat-killed *Salmonella typhimurium, Listeria monocytogenes,* and Mycobacterium marinum did not elicit a protective effect against a subsequent lethal challenge of the same bacteria used in the priming dose (Figures 2A and S2). Dead bacteria were used as priming doses in these experiments because all of these bacteria have an LD_50_ of one bacterium. Protection by priming is thus not a general characteristic of all microbial challenges in the fly. However, a priming dose of the natural fungal pathogen, Beauveria bassiana [17], conferred a protective effect against a subsequent lethal challenge (Figures 2A and S2).

Perhaps S. pneumoniae is a uniquely powerful immune activator; priming with S. pneumoniae might protect against challenges with other pathogens. To test this hypothesis, we challenged S. pneumoniae–primed flies with lethal doses of the same panel of microbes as above. S. pneumoniae–primed flies were not protected against lethal challenges of other pathogens (Figures 2A and S2). The protective effect of the primed response thus is not due to general activation of the *Drosophila* immune response.

Having seen that the priming-induced protective response was specific in the sense that S. pneumoniae was incapable of protecting against other immune challenges, we next determined whether other immune activators were capable of inducing a protective response against *S. pneumoniae.* To test this, we primed flies with a mixture of strong immune activators (dead *Escherichia coli,* dead *Micrococcus luteus,* and dead Beauveria bassiana) [17,19–21]. Flies injected with this mixture were not protected against a lethal challenge of S. pneumoniae (Figure 2B). Furthermore, injection with this mixture does not interfere with the ability to induce a protective response because the addition of dead S. pneumoniae to the mixture protected the flies from a second lethal challenge (Figure 2B). These results also demonstrate that a priming dose of B. bassiana does not protect against a lethal dose of S. pneumoniae. We conclude that protection conferred by a priming dose of S. pneumoniae specifically protects against lethal doses of S. pneumoniae and persists for the life of the fly.

### The Toll Pathway, but Not the imd Pathway, Is Required for the Primed Response to S. pneumoniae

**Figure 3:**
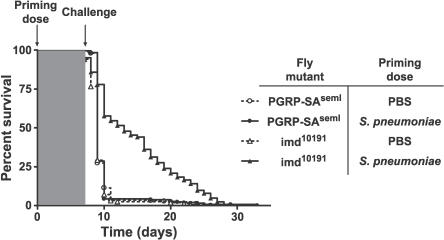
The Toll Pathway Is Required for the Primed Response Partial loss-of-function alleles of PGRP-SA (circles) and imd (triangles) were injected with PBS (open shapes) or dead S. pneumoniae (filled shapes) on day 0 and challenged 1 wk later with a lethal dose of S. pneumoniae (20 CFU for PGRP-SA^seml^ and 100 CFU for imd^10191^). Log-rank analysis of the survival curves indicates that naïve and S. pneumoniae–primed PGRP-SA^seml^ flies die at the same rate, whereas the curves for naïve and S. pneumoniae–primed imd^10191^ flies are significantly different (*p* < 0.0001). *n* = 154 to 245 for each condition. Molecular information for imd^10191^ is given in Materials and Methods.

Toll and imd are the best-characterized fly immunity pathways that control the majority of genes found to be induced by bacterial and fungal infections, including AMPs [1,2,17,19–21]. The mixture of microbes used in Figure 2B was chosen to strongly activate both the Toll and imd pathways [19,20]. Although activation of Toll and imd signaling is insufficient to induce a protective response, it remained possible that the pathways are necessary for protection. We tested loss-of-function mutants in each pathway to determine whether they were necessary for a protective response. Because both Toll and imd pathway mutants are immunocompromised with respect to S. pneumoniae (Figure S3), we reduced the lethal challenge dose of S. pneumoniae (20 CFU for Toll mutants, 100 CFU for imd mutants). These doses are normally sublethal to wild-type flies, but higher doses killed the mutant flies too quickly to detect a difference in survival. Loss-of-function mutants of imd (Figure 3) and dTak1 [22] (unpublished data) were protected by a priming dose of dead S. pneumoniae. Thus, although the imd pathway normally contributes to killing *S. pneumoniae,* it is not necessary to elicit a protective response. In contrast, flies homozygous for partial loss-of-function mutations that disrupt the Toll pathway, PGRP-SA [23,24] (Figure 3) and Dif [25] (unpublished data), were not protected by a priming dose of dead *S. pneumoniae.* The Toll pathway is therefore necessary for the primed response, but the imd pathway is not required.

### Phagocytes Are the Critical Effectors of the Primed Response

**Figure 4:**
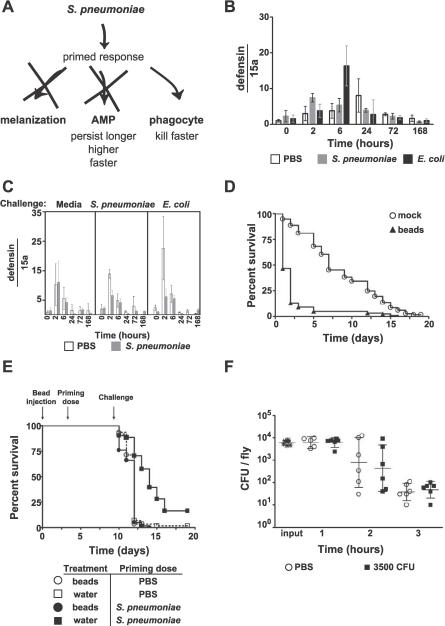
S. pneumoniae–Primed Flies Exhibit an Enhanced Phagocytic Response That Is Specific to S. pneumoniae (A) Model for S. pneumoniae–induced primed response. (B and C) RNA was extracted from whole flies. Defensin transcript levels were quantified using qRT-PCR and normalized to 0-h media injection. Bars represent mean values with standard deviation. See Figures S4 and S5 for diptericin and attacin transcript levels. (B) Flies were injected with media (open bars), 250 CFU of S. pneumoniae (gray bars), or 250 CFU of E. coli (black bars). (C) Flies were primed on day 0 with media (white bars) or dead S. pneumoniae (gray bars) and challenged 1 wk later with media, 3,500 CFU of S. pneumoniae, or 3,500 CFU of E. coli (indicated above the graph). (D) Flies were injected with polystyrene beads (triangles, *n* = 122) or water (circles, *n* = 115) 3 d prior to day 0 to fully inhibit phagocytosis. On day 0, flies were injected with 20 CFU of S. pneumoniae. Bead-inhibited flies die significantly faster than do mock-injected flies (*p* < 0.0001, log-rank analysis of survival curves). (E) Flies were injected with polystyrene beads (circles) or water (squares) on day 0, injected with a priming dose of PBS (open shapes) or S. pneumoniae (filled shapes) on day 3, and injected with a lethal dose of 1,000 CFU on day 10. Log-rank analysis of the survival curves indicates that all flies died significantly faster than did mock-treated S. pneumoniae–primed flies (*p* < 0.0001). *n* = 51 to 62 for each condition. (F) Flies were injected with PBS (circles) or dead S. pneumoniae (squares) on day 0 and challenged 1 wk later with 6,000 CFU of E. coli. Bars represent geometric means of bacterial load with 95% confidence intervals.

Next, we wanted to determine the contribution of melanization, the humoral response, and the cellular response to priming (Figure 4A) [1,2]. We do not observe melanization in response to S. pneumoniae infections and therefore do not expect that it plays a strong role in the protective response (unpublished data) [26]. Because the Toll pathway is required for the priming-induced protection, we first examined the contribution of the inducible humoral response.

We found four lines of evidence suggesting that AMPs are not responsible for protecting flies against a second lethal challenge of *S. pneumoniae.* First, S. pneumoniae was not a strong AMP inducer because the peak of AMP transcription in response to S. pneumoniae was not as high as the peak induced by the positive control elicitor E. coli [27]. Flies were injected with a priming dose of dead *S. pneumoniae,* media (wounding control), or E. coli (positive control), and quantitative real-time reverse transcription–PCR (qRT-PCR) was used to assess transcript levels of three different AMPs: defensin (Figure 4B), attacin (Figure S4A), and diptericin (Figure S4B) [27]. These AMPs were chosen because they are strongly induced by Gram-positive bacterial infections. Second, we found that not only was S. pneumoniae a poor inducer of AMPs but also AMP transcript levels did not remain elevated 1 wk later (Figure 4B). Thus, AMP transcription should be back to the ground state by the time the challenge dose is administered, 1 wk after the priming dose. It has been reported that AMPs can persist in the hemolymph [28]. However, we have shown that simultaneous activation of Toll and imd, and thus AMP induction, is not sufficient to protect the fly (Figure 2B). Finally, we asked if this ground state is “sensitized”—that is, whether AMP induction is enhanced in flies that have been primed with S. pneumoniae compared to media. Using qRT-PCR, we measured defensin (Figure 4C), attacin (Figure S5A), and diptericin (Figure S5B) transcript levels after a second challenge of S. pneumoniae, media (wounding control), or E. coli (positive control). None of the AMPs were differentially induced in S. pneumoniae–primed flies compared to naïve media-injected flies. Thus, we find no evidence to support the involvement of AMP induction in the primed immune response.

In light of these data, and the fact that hemocytes are altered in Toll pathway mutants [12,13], we examined whether the cellular immune response is the main effector of the primed response—that is, a priming dose of S. pneumoniae might specifically increase S. pneumoniae clearance by phagocytes upon a second exposure. We first assessed the contribution of phagocytosis to S. pneumoniae killing in naïve flies by injecting flies with polystyrene beads to block phagocytosis prior to infection [29]. Bead-inhibited flies were extremely sensitive to *S. pneumoniae;* 3,000 bacteria were required to kill a wild-type fly, whereas 20 CFU was sufficient to kill a wild-type fly that lacks phagocytosis (Figure 4D). We demonstrated above that the survival difference between S. pneumoniae–primed flies and naïve flies is linked to enhanced clearance (Figure 1D); here we show that phagocytosis is required to kill S. pneumoniae in naïve flies.

We then asked if the enhanced clearance in primed flies is due to increased killing by phagocytes and not a second, mechanistically, different method of killing. To test this, we inhibited phagocytosis in both primed and naïve flies and then challenged with a lethal dose of S. pneumoniae 1 wk later, at which time point phagocytosis remained inhibited [29]. Primed flies died at the same time as naïve flies and were therefore not protected by a priming dose of *S. pneumoniae,* regardless of whether they were injected with beads before (Figure 4E) or after (unpublished data) the priming dose. Fly phagocytes are therefore an essential effector of the primed response.

These data suggested that a priming dose of S. pneumoniae activates fly phagocytes to kill S. pneumoniae more efficiently. Is this enhanced killing specific to *S. pneumoniae,* or does a priming dose of S. pneumoniae simply cause general phagocyte activation? If phagocytes are generally more activated in S. pneumoniae–primed flies, these flies should be able to clear other bacteria more rapidly. To test this hypothesis, S. pneumoniae–primed flies were tested for their ability to kill E. coli (Figure 4F). There was no difference between naïve PBS-injected and S. pneumoniae–primed flies in their ability to clear *E. coli.* Combined with the fact that a priming dose of S. pneumoniae does not offer protection against any other lethal challenges of bacteria (Figure 2A) and the fact that the primed response is specific for S. pneumoniae (Figure 2), we conclude that a priming dose of S. pneumoniae alters the fly immune system in a persistent manner that specifically allows phagocytes to recognize and kill S. pneumoniae more efficiently.

### Concluding Remarks

We have presented evidence that the fly modulates its immune response as a result of multiple challenges: a priming dose of S. pneumoniae is sufficient to protect the fly against a subsequent lethal dose of *S. pneumoniae.* Using a functional immune assay, we have shown that the fly immune system exhibits the adaptive characteristics of specificity and persistence. The mechanism underlying this protective response requires the Toll pathway, although its contribution is not via activation of AMPs. We have eliminated contributions from the imd pathway and AMPs and identified phagocytes as the critical effectors of the primed response. This system is uniquely positioned to further characterize the molecular basis underlying specific phagocyte activation and other adaptive aspects of innate immunity.

## Materials and Methods

### Fly stocks.

Flies were maintained on standard dextrose medium at 25 °C and 65% humidity. All experiments were performed with 5- to 7-d-old male wild-type Oregon R flies. All mutant flies were back-crossed onto the Oregon R background to limit background effects. In particular, white mutant flies are very sensitive to S. pneumoniae and do not elicit a primed response (unpublished data). Mutant lines used in this study include PGRP-SA^seml^ (from P. Ligoxygakis), Dif, imd^10191^, and Tak1^2527^. Molecular information for imd^10191^ is included below.

### Molecular information on imd^10191^.

The imd^10191^ line has a 26-nucleotide deletion that frameshifts the protein at amino acid 179, which is the beginning of the death domain.

### Microbial strains and culture.

Microbial strains used in this study include S. pneumoniae strain SP1, E. coli DH5α, *M. luteus, B. bassiana*, L. monocytogenes strain 10403S, S. typhimurium strain SL1344, and M. marinum strain M. S. pneumoniae cultures were grown standing at 37 °C 5% CO_2_ in brain heart infusion broth (BHI) (BD Bioscience, http://www.bdbioscience.com) to an OD_600_ of 0.15, and aliquots were frozen at −80 °C in 10% glycerol. For infection, an aliquot of S. pneumoniae was thawed, diluted 1:3 in fresh BHI, and allowed to adjust for 2 h at 37 °C 5% CO_2_. *E. coli, S. typhimurium,* and L. monocytogenes cultures were grown standing overnight in BHI at 37 °C. M. luteus was cultured standing at 29 °C in BHI for 1 wk or until a sufficient density was reached. M. marinum was cultured standing at 29 °C in Middlebrook 7H9 broth (BD Bioscience) supplemented with Middlebrook OADC (BD Bioscience) and 0.2% Tween. B. bassiana spores were grown on malt agar (BD Bioscience) at 29 °C for 2 wk or until a sufficient density was reached.

### Fly injections.

For injection, flies were anesthetized with CO_2_ and injected with a total volume of 50 nl using individually calibrated pulled glass needles attached to a Picospritzer III injector (Parker Hannifin, http://www.parker.com). Flies were always injected in the abdomen, close to the junction with the thorax and just ventral to the junction between the ventral and dorsal cuticles. Flies were never anesthetized for longer than 10 min. After each injection, all flies were transferred to a new vial and maintained at 29 °C and 65% humidity.

### Priming doses of microbes.

To prepare priming doses of microbes, concentrated cultures were boiled for 30 min, centrifuged 5 min at 2,000*g*, and washed three times in PBS. Bacterial cultures were diluted in PBS to an OD_600_ of 0.1 and stored at −80 °C. Heat-killed B. bassiana spores were counted on a hemocytometer, adjusted to a concentration of 1 × 10^7^/ml in PBS, and stored at −80 °C. Aliquots were plated on the appropriate media to verify that the microbes had been heat-killed. To prime flies, 50 nl of these solutions was injected into the fly. Flies were incubated at 29 °C and 65% humidity until they received their second challenge.

### Lethal challenges of microbes.

Fresh cultures were washed three times in PBS and diluted to the appropriate OD_600_ in PBS. For the different concentrations of *S. pneumoniae,* appropriate bacterial load corresponding to different optical densities was experimentally determined. For reference, an OD_600_ of 0.1 corresponds to 3,000 CFU. Lethal doses of other bacterial species are as follows: *S. typhimurium,* OD 0.1 (10,000 CFU); *L. monocytogenes.* OD 0.01 (6,500 CFU); and *M. marinum,* OD 0.05 (500 CFU). Bacterial load after injection was verified for all strains except M. marinum by plating on the appropriate media (blood agar for S. pneumoniae, BHI agar for all other strains). For lethal B. bassiana challenges, flies were anesthetized in groups of 20 and shaken on a plate of spores for exactly 30 s. All infections were carried out at 29 °C.

### Determination of CFU in flies.

Individual flies were homogenized in 100 μl of PBS, diluted serially, and spotted onto appropriate plates. S. pneumoniae were grown on blood agar supplemented with 500 μg/ml streptomycin (Sigma, http://www.sigmaaldrich.com), 10 μg/ml colistin (Sigma), and 5 μg/ml oxolinic acid (Sigma) to eliminate the growth of bacterial contaminants from the fly. Plates were incubated overnight at 37 °C 5% CO_2_. E. coli colonies were grown on LB and incubated overnight at 37 °C.

### qRT-PCR.

Flies were challenged as described above and incubated at 29 °C for the indicated time points. At the given times, triplicates of three flies were anesthetized, placed in 1.5-ml tubes, and homogenized in 100 μl of Trizol-LS (Invitrogen, http://www.invitrogen.com). RNA was extracted using the standard Trizol-LS protocol, and remaining genomic DNA was degraded with DNase I treatment. RT-PCR was carried out with a Bio-Rad iCycler (http://www.bio-rad.com) using TaqMan probes and rTth polymerase (Perkin-Elmer, http://www.perkinelmer.com) as directed by the manufacturer. The following primers below were used. Relative RNA quantities were determined with respect to *Drosophila* ribosomal protein 15a, and all levels were normalized with respect to the zero time point for media injection: defensin TTCTCGTGGCTATCGCTTTT (left primer), GGAGAGTAGGTCGCATGTGG (right primer), AGGATCATGTCCTGGTGCATGAGGA (Taqman probe); attacin CAATGGCAGACACAATCTGG (left primer), ATTCCTGGGAAGTTGCTGTG (right primer), AATGGTTTCGAGTTCCAGCGGAATG (Taqman probe); diptericin ACCGCAGTACCCACTCAATC (left primer), CCCAAGTGCTGTCCATATCC (right primer), CAGTCCAGGGTCACCAGAAGGTGTG (Taqman probe); and ribosomal protein 15a TGGACCACGAGGAGGCTAGG (left primer), GTTGGTTGCATGGTCGGTGA (right primer), TGGGAGGCAAAATTCTCGGCTTC (Taqman probe).

### Bead injections.

Carboxylate-modified blue fluorescent 0.2-μm-diameter polystyrene beads (Molecular Probes, http://www.invitrogen.com) were injected to block phagocytosis essentially as previously described [29]. Briefly, beads were washed twice in sterile water and resuspended in one fourth of the original volume. Flies were injected with 50 nl of bead solution or water as an injection control. To confirm that phagocytosis was inhibited, the in vivo phagocytosis assay was performed as described previously with FITC-conjugated E. coli or FITC-conjugated *Staphylococcus aureus.* Phagocytic inhibition was confirmed each time bead-injected flies were manipulated.

### Data analysis.

All experiments were performed at least three times. For survival analysis, a minimum of 45 flies were injected for each condition. Dead flies were counted daily, and survival data were graphed and analyzed using GraphPad Prism (GraphPad Software, http://www.graphpad.com). Mean survival time with standard error was calculated using R (http://www.r-project.org).

## Supporting Information

Figure S1 S. pneumoniae–Induced Protection Persists for the Life of the Fly Flies were primed on day 0 with PBS (open circles) or dead S. pneumoniae (filled squares). Flies were challenged with 5,000 CFU on the indicated days, and log-rank analysis was performed. A representative experiment (of three replicates) is shown. (A) Day 1, *p* < 0.0001; (B) day 3, *p* = 0.0038; (C) day 7, *p* < 0.0001; (D) day 10, *p* = 0.0037; (E) day 14, *p* = 0.0003.
(89 KB PDF)

Figure S2 Specific Protection Elicited by Priming Doses of S. pneumoniae and B. bassiana
Flies were injected with a priming dose of PBS (open circles), the same bacteria used for the lethal challenge (triangles) or S. pneumoniae (boxes). One week later, flies were injected with the lethal challenge listed, and log-rank analysis was performed (statistics shown below the graph; ns = not significant). A representative experiment (of three replicates) is shown. (A) *S. typhimurium;* (B) *L. monocytogenes;* (C) *M. marinum;* (D) *B. bassiana;* (E) *S. pneumoniae.*
(154 KB PDF)

Figure S3 The Toll and imd Pathways Contribute to S. pneumoniae Killing Wild-type (squares, 5,000 CFU, *n* = 184) and partial loss-of-function alleles of imd (triangles, 100 CFU, *n* = 180) and PGRP-SA (circles, 20 CFU, *n* = 177) were injected with lethal doses of *S. pneumoniae.* The curves corresponding to imd and PGRP-SA differ significantly from wild-type at *p* < 0.0001 (log-rank analysis).
(20 KB PDF)

Figure S4 AMP Transcription Is Not Strongly Induced by S. pneumoniae and Does Not Persist for 1 wk Flies were injected with media (open bars), 250 CFU of S. pneumoniae (gray bars), or 250 CFU of E. coli (black bars). RNA was extracted from whole flies. Attacin (A) and diptericin (B) transcript levels were quantified using qRT-PCR and normalized to 0-h media injection. Bars represent mean values with standard deviation.
(185 KB PDF)

Figure S5 AMPs Are Not Differentially Induced in S. pneumoniae–Primed Flies Flies were injected with a priming dose of media (white bars) or dead S. pneumoniae (black bars) and challenged 1 wk later with media, 3,500 CFU of *S. pneumoniae,* or 3,500 CFU of E. coli (indicated above the graph). RNA was extracted from whole flies. Attacin (A) and diptericin (B) transcript levels were quantified using qRT-PCR and normalized to 0-h media injection. Bars represent mean values with standard deviation.
(223 KB PDF)

## Abbreviations

AMP: antimicrobial peptide
CFU: colony-forming units
PBS: phosphate-buffered saline
qRT: quantitative real-time reverse transcription
