# Supplementary material for: A Specific Primed Immune Response in Drosophila Is Dependent on Phagocytes
Source: PLoS Pathog. 2007 Mar 9;3(3):e26. doi: 10.1371/journal.ppat.0030026 (PMC1817657; doi:10.1371/journal.ppat.0030026)

Supplemental Figure 4 Pham et al.

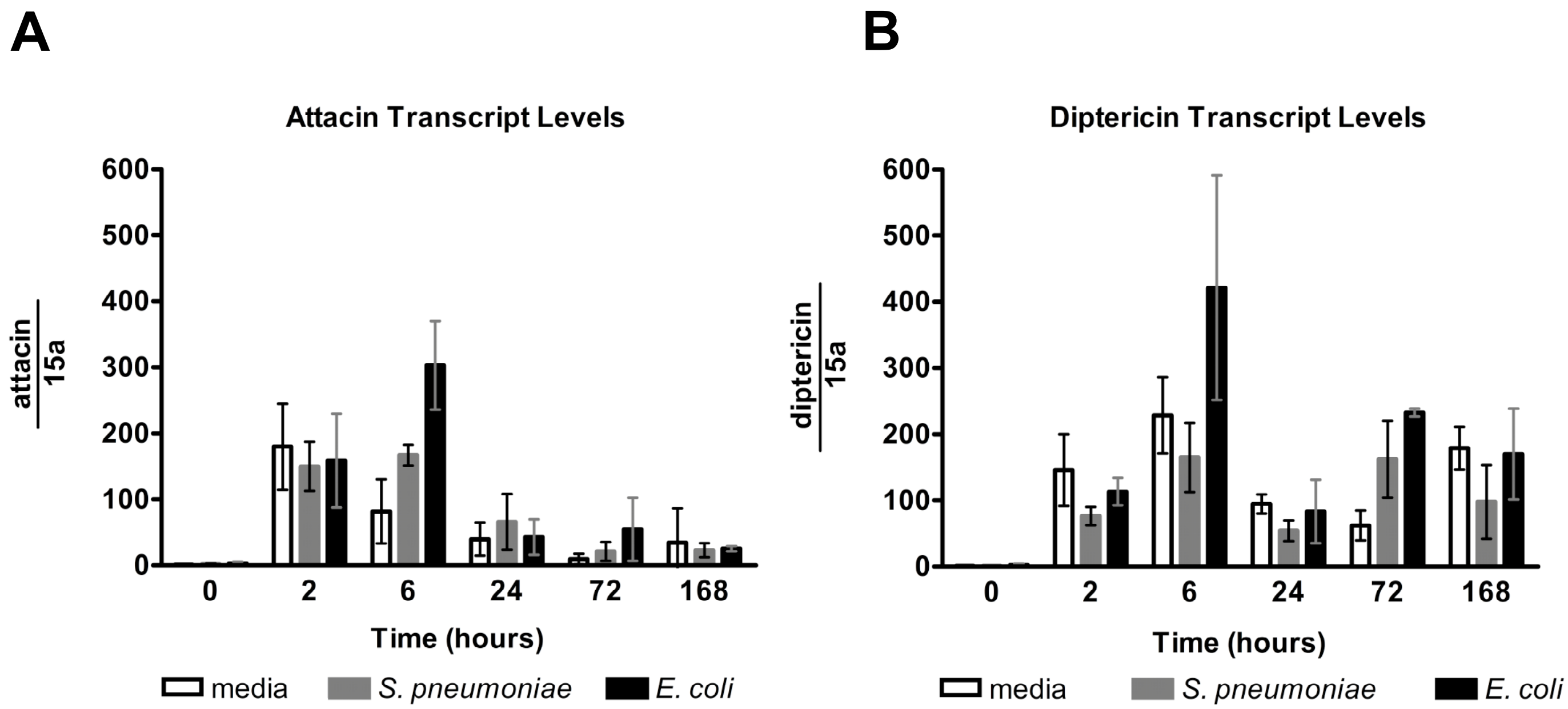

Supplement: Supplementary file 4 [file ppat.0030026.sg004.pdf]
